# Out-of-hours services in Zealand, Denmark. Consequences of changeover from GP-cooperative to integrated deputized services. A retrospective cohort study

**DOI:** 10.1080/02813432.2026.2616519

**Published:** 2026-02-07

**Authors:** Stig Nikolaj Fasmer Blomberg, Hauraz Haji, Ole Mazur Hendriksen, Mai-Britt Hägi-Pedersen, Helle Collatz Christensen

**Affiliations:** ^a^ Prehospital Centre, Region Zealand, Denmark; ^b^ Department of Health Science and Technology, Aalborg University, Cardiotech, Denmark; ^c^ Primary care and E-hospital, Region Zealand, Denmark; ^d^ University College Absalon, Denmark; ^e^ University of Copenhagen Institute of Clinical Medicine, Copenhagen, Denmark

**Keywords:** Emergency medical services, out-of-hours services, Region Zealand, general practitioners, healthcare facilities

## Abstract

**Objectives:**

Out-of-hours services (OOHS) worldwide exhibit diverse organisational models, especially within the European Union. This study aims to describe the transformation of OOHS in Region Zealand, Denmark, from a General Practitioner cooperative (GP-OOHS) to a regional organization/service, known as the 1818 Medical Helpline (1818).

**Study Design:**

Retrospective cohort study.

**Methods:**

GP-OOHS data (January 2017–October 2023) during the transition from GP-cooperative to Regional Service (October 2022) were analysed. Coded and timestamped services retrieved from the billing system were used to identify patient pathways, defined as services within a 12-hour window. Descriptive statistics were applied.

**Results:**

A total of 2,572,951 patient pathways were recorded, with 2,258,072 under GP-out-of-hours service and 314,879 under the 1818. Overall, patient pathway volumes declined from 412,116 in 2017 to 314,879 in 2022, and admissions fell from 64,555 to 59,967. The median patient age was 35 years. The GP-out-of-hours service had a higher average monthly volume of patient pathways (32,726 vs. 25,940), while 1818 showed a higher proportion of emergency department admissions within 24 h (19.0% vs. 17.6%), the number of admissions fell from 8.2 per 100,000 inhabitants to 7.1 per 100,000 inhabitants. Interrupted time series analysis showed that the previously increasing tendency in ED admissions flattened following the transition. Although the absolute number of children admitted decreased, the proportion of admissions increased due to overall decline in patient pathways. Face-to-face consultations (31.4% vs. 27.1%) and home visits (9.4% vs. 3.4%) were more frequent in GP-out-of-hours service, both being associated with higher admission rates.

**Conclusions:**

OOHS patient pathways declined over the study period and continued to decline following the organizational change. The absolute number of emergency department admissions also decreased, while the relative proportion of admissions increased. These findings indicate a continued decline in OOHS activity and a slight shift toward more acute cases after the reorganisation.

## Introduction

Out-of-hours services (OOHS) vary in their organizational models worldwide, reflecting national variations in healthcare organization, workforce capacity, and service delivery models [1–3]. Within the European Union, countries typically operate between three and 10 coexisting models, with general practitioner (GP) cooperatives being the most common, followed by primary care centres and rota groups of GPs [4,5]. Recent trends in OOHS include organizational restructuring and service upscaling [6]. These developments reflect a broader shift towards centralization, optimization, professionalization, and expanded diagnostic capabilities. However, these national variations in OOHS organization underscore regional disparities in access and raise concerns about potential care fragmentation.

The Danish healthcare system provides emergency medical services (EMS) and out-of-hours care for medical problems arising outside regular working hours [1,7]. Until October 2022, OOHS in Region Zealand’s were provided by GP cooperatives. These services were staffed by GPs working outside regular office hours. However, increasing difficulty in maintaining on-call coverage, driven by physician shortages and healthcare centralization prompted the region to implement a new integrated service model.

The new system, known as ‘1818’ (after the designated phone number), employs physicians of any specialty directly to triage all calls and compensates them on an hourly basis. To enhance flexibility and efficiency nurses were introduced during peak times. Although physical service locations remained unchanged, the payment model was altered: GPs was employed by the region and paid on hourly basis. Before implementation, emergency departments (ED), already understaffed and overburdened - expressed concern that the reorganisation might increase ED activity, as had been observed in the neighbouring Capital Region after a similar reform in 2014 [15].

The characteristics of the two types of organisations are shown in Table 1.

**Table 1.** Comparison of previous GP-OOHS and current 1818 in Zealand Region.

Domain	Previous model (GP cooperative, Region Zealand, before October 2022)	Current model (1818 OOHPC, Region Zealand, from October 2022)
Organizational structure	Regional GP cooperative organized as part of general practice out-of-hours system	Single regional out-of-hours primary care (OOHPC) service operated directly by Region Zealand
Service name	GP-OOHS	1818 Medical Helpline (named after the hotline number)
Contact telephone number	70 15 07 00	1818
Access to care	Patients contacted GP-OOHS directly by telephone	Patients contact regional 1818 directly by telephone
Service hours	Weekdays 4 PM–8 AM; weekends and public holidays 24/7	Weekdays 4 PM–8 AM; weekends and public holidays 24/7
Triage personnel	Telephone triage performed exclusively by GPs	Telephone triage performed by physicians from any medical specialty and nurses
Use of decision support	Based on GP clinical expertise and judgment; no formal decision support tools	No decision support tools; nurses follow predefined clinical instructions under delegated medical responsibility
Remote consultations	Telephone consultations provided by GPs; no systematic use of video	Telephone and video consultations provided by physicians and nurses
In-clinic consultations	All consultations conducted by GPs	Consultations conducted by physicians with nurse assistance; nurses may complete consultations after physician assessment
Home visits (16:00–22:00)	Home visits performed by GPs	Physicians perform home visits accompanied by paramedics
Home visits (22:00–08:00)	Home visits performed by GPs	Paramedics perform home visits with telephone supervision by EMCC* physician
Physical locations	Several fixed consultation sites, mostly located on hospital premises	Physical clinic locations unchanged
Diagnostics available	Limited point-of-care testing in clinics; minimal diagnostics during home visits	Not specified; likely limited diagnostics
Remuneration model	Fee-for-service payment to GPs	Fixed hourly wages for all staff
Medical record accessibility	Records not integrated with hospital records; separate GP-OOHS system (Medwin)	OOHPC records accessible to hospital staff

* Emergency Medical Coordination Centre.

This retrospective cohort study investigates the impact of the reorganization on ED admissions. We hypothesized that the transition would result in higher ED admission rates as seen in The Capital Region. Secondary outcomes included differences in overall activity between the two service models.

## Methods

### Setting

Region Zealand is a mixed rural-urban region in Denmark with 850,000 inhabitants, where Out-of-hours medical needs are met by regionally organized services outside standard GP opening hours.

The OOHS operates through telephone triage, where patients’ needs are assessed and results in one of several outcomes: telephone advice, video-consultation, physical consultation, home visit, or ED admission. Patients are free to choose any OOHS facility and may be booked for the consultation that best fits their location and preferences. OOHS is available from 4:00 PM to 8:00 AM on weekdays and all hours on weekends and public holidays [7,8].

### Data collection

Administrative and billing data were obtained from both the GP-OOHS and the 1818. The dataset had information on the type of service delivered (code), patients’ ID and time of service delivery. The data structure was uniform during the entire study period, with no changes to the electronic health record system.

All services provided by GP-OOHS or 1818 in the dataset are given a code and a timestamp.e.g. ‘0101 Consultation’, ‘0301 Telephone Consultation’, ‘0471 Home Visit’ etc. For each patient pathway, all codes were stored with a timestamp for the service, and the patient’s personal identifier [9]. Data were collected for the period between January 1, 2017, and October 1, 2023. The transition from independent GP-OOHS to 1818 was effective from October 1, 2022.

**Figure 1.**
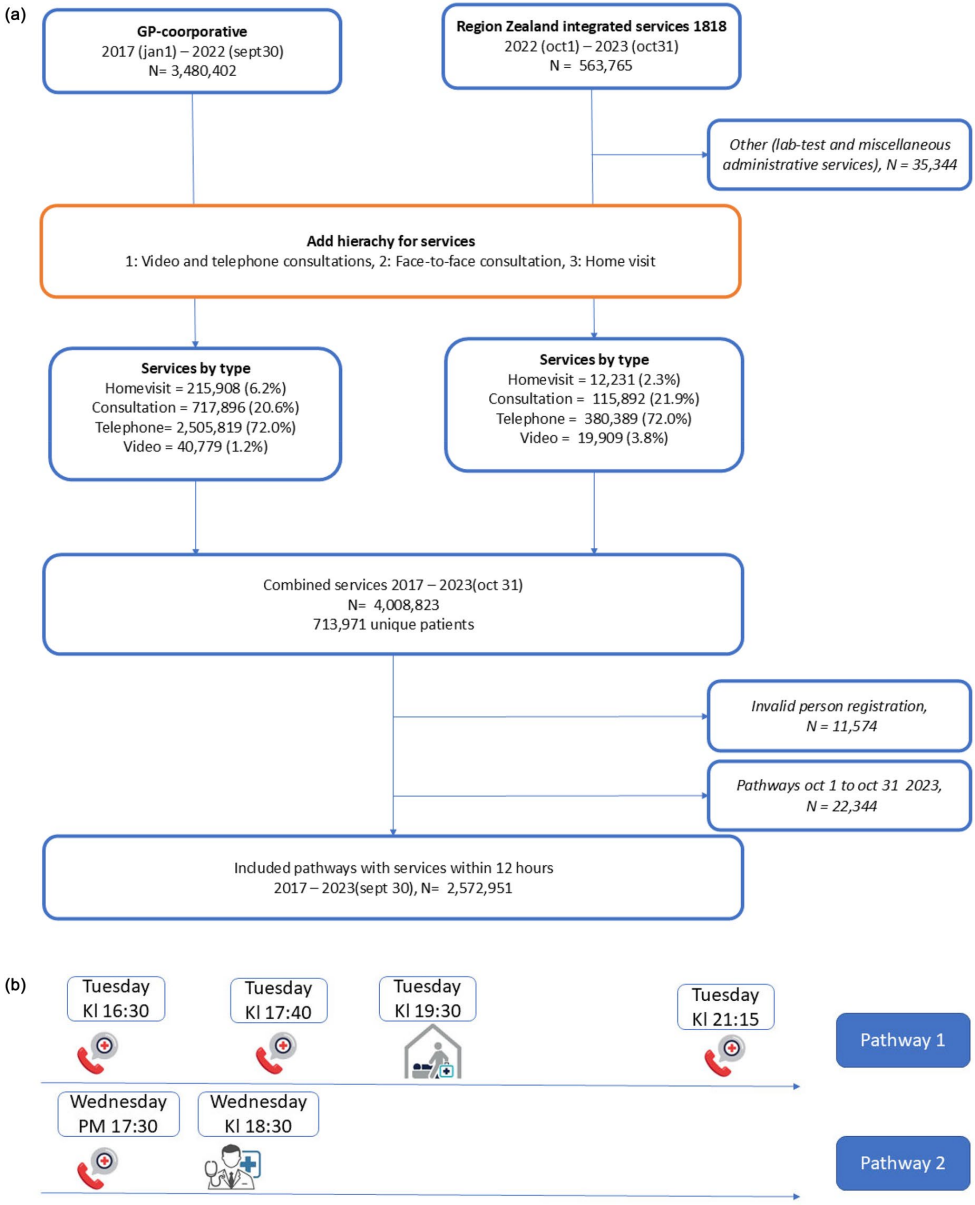
a. Population flowchart, Out-of-hours-services in Region Zealand. b. Patient pathways in Out-of-hours-services in Region Zealand.

Defining Patient Pathways: In this study, we define a *patient pathway* as a sequence of one or more services delivered within a 12-hour window. Each patient pathway reflects a single episode of acute care and includes all OOHS interactions (calls, consultations, visits) during that time frame. The 12-hour window was chosen as it corresponds to the typical period until general practices reopen the following day. This definition allows us to analyse the number of distinct acute episodes requiring out-of-hours care, rather than the number of individual service interactions. (See Figure 1(b)).

The term *patient pathway* is used throughout the manuscript to denote these grouped episodes of care. While the underlying dataset consists of timestamped service codes (e.g. phone calls, home visits), our analyses focus on patient pathways as the unit of analysis.

This grouping was chosen to reflect the clinical reality of acute illness episodes rather than individual service events. By treating multiple services within a 12-hour window as a single patient pathway, we avoid overcounting patients who may, for example, call several times in the same episode of illness. For the purposes of this study, the key unit of analysis is not the number of service interactions, but the number of distinct patient episodes that triggered OOHS involvement. This is particularly important when assessing downstream outcomes such as ED admission. Using individual services as the denominator would give a misleading picture of the admission rate, as some patients, especially those with more complex or uncertain symptoms, might generate multiple entries for what is in practice a single clinical situation. For each patient pathway, the time of the first and last service were recorded, as well as the service with the highest clinical hierarchy. Telephone and video consultations were ranked lowest, followed by face-to-face consultations, and home visits ranked highest. Thus, in the example described above, the first patient pathway would be categorized as ending in a home visit.

Linking to ED Admissions: Once the dataset of patient pathways was created, we added data on ED admissions. ED admissions were identified by linkage through the unique personal identification number (CPR) to the Danish National Patient Register (LPR) [9,10]. Any admission occurring within 24 h after the last recorded service was categorized as a ED admission using a binary variable.

For trend analyses, we restricted the dataset to the period October 2017–October 2023 to allow for consistent year-over-year comparisons. The first five years (October 2017–October 2022) correspond to the GP-OOHS model, and the final year (October 2022–October 2023) corresponds to the 1818.

## Statistics

Descriptive analyses were performed, and absolute numbers and percentages for variables were reported. To assess changes over time and the potential impact of the organisational transition to the 1818 (October 2022), an interrupted time series (ITS) regression analysis was conducted based on aggregated 12-month periods (October–September). The models estimated pre-intervention trends and post-intervention level changes for both pathway counts (Poisson regression) and admission proportions (binomial regression). The data analyses and management were conducted using R (version 3.5.3, R Core Team), and SAS statistical software (version 9.4, SAS Institute).

## Results

Between 2017 and October 2023, a total of 2,572,951 patient pathways were recorded (Table 2). The median patient age was 35 years in both organizations, with slight variation in age distribution: in GP-OOHS, the interquartile range was 15–59 years, while in 1818 it was 14–62 years. The GP-OOHS handled a higher average monthly volume of 32,726 patient pathways compared to 26,240 patient pathways per month in 1818.

**Table 2.** Comparison of patient pathways and demographics between GP-OOHS and 1818 and the combined total of both models.

	GP-OOHS, *n* = 2,258,072	1818, *n* = 314,879	TOTAL, *n* = 2,572,951
Age, Median (Q1;Q3)	35 (15;59)	35 (14;62)	35 (15;60)
Male %, (n)	44.5 (1,004,821)	44.3 (139,370)	44.5 (1,144,191)
Patient pathways per month (average)	32,726	26,240	31,765
Proportion ended in phone, %	57.9	64.7	58.7
Proportion ended in face-to-face consultation %	31.4	27.0	30.9
Proportion ended in homevisit, (%)	9.4	3.3	8.6
Proportion ended in videoconsult, (%)	1.4	5.0	1.8
Age 0–17, % (n)	27.8 (627,753)	28.7 (90,373)	27.9 (718,126)
Age 18–65, % (n)	52.3 (1,182,606)	49.6 (156,155)	52.0 (1,338,761)
Age 66–85, % (n)	15.8 (355,990)	17.3 (54,421)	16.0 (410,411)
Age +85, % (n)	4.1 (91,723)	4.4 (13,930)	4.1 (105,653)
Average number of individuals/month	28.005	23.248	27.224
Average number of patient pathways/month	32.726	26.240	31.765
Weekday, % (n)	47.4 (1,069,856)	47.8 (150,370)	47.4 (1,220,226)
Call 08–15, % (n) (weekends/ holidays only)	30.3 (683,816)	31.,0 (97,528)	30.4 (781,344)
Call 16–22, % (n)	54.6 (1,232,119)	52.2 (164,380)	54.3 (1,396,499)
Call 23-07, % (n)	15.2 (342,137)	16.8 (52,971)	15.4 (395,108)

The table highlights distinct differences in patient management and service delivery between the GP-OOHS and 1818. The GP-OOHS handles a higher volume of patient pathways and conducts more face-to-face consultations and home visits, whereas the 1818 has a greater reliance on phone and video consultations. The age distribution shows a slightly higher proportion of elderly patients in GP-OOHS. The time interval “08–15” refers exclusively to weekends and holidays. Overall, the data reflects variations in consultation practices, patient demographics, and contact timings between the two service models.

**Figure 2.**
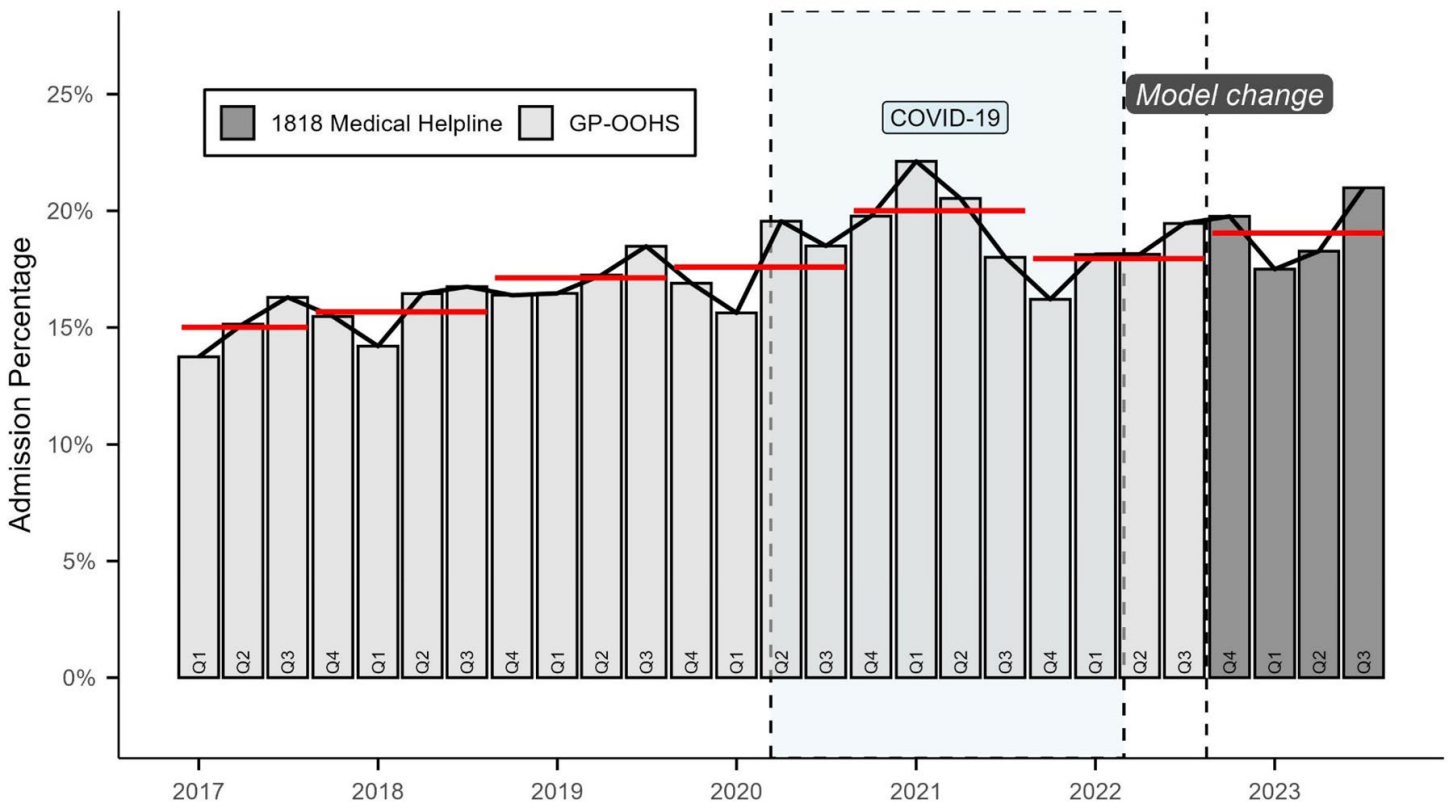
Temporal volume of consultations resulting in admission. This figure illustrates the temporal trend (percentage of quarterly cases) admitted to emergency department during the study period (2017–2023). The bars in light grey (Q1 2017 to Q3 2022) represent the period with patient management using the GP-OOHS, whereas the dark grey bars (Q4 2022 to Q3 2023) represent the period with patients managed by 1818. The dashed vertical line marks the transition between the models. The horizontal red lines showcase the median admission percentage prior to the transition (GP-OOHS) and one year after (1818).

Seasonal variation was comparable between the two models, although GP-OOHS consistently maintained a higher level of activity (Figure 2).

Face-to-face consultations were more frequent in GP-OOHS (31.4%) than in 1818 (27.0%). Similarly, home visits were more common in GP-OOHS (9.4% vs. 3.3%). Conversely, telephone consultations accounted for a larger share of patient pathways in 1818 (64.7%) compared to GP-OOHS (57.9%). Video consultations were introduced later in GP-OOHS (2021), which explains their lower overall share (1.4% vs. 5.0%).

**Figure 3.**
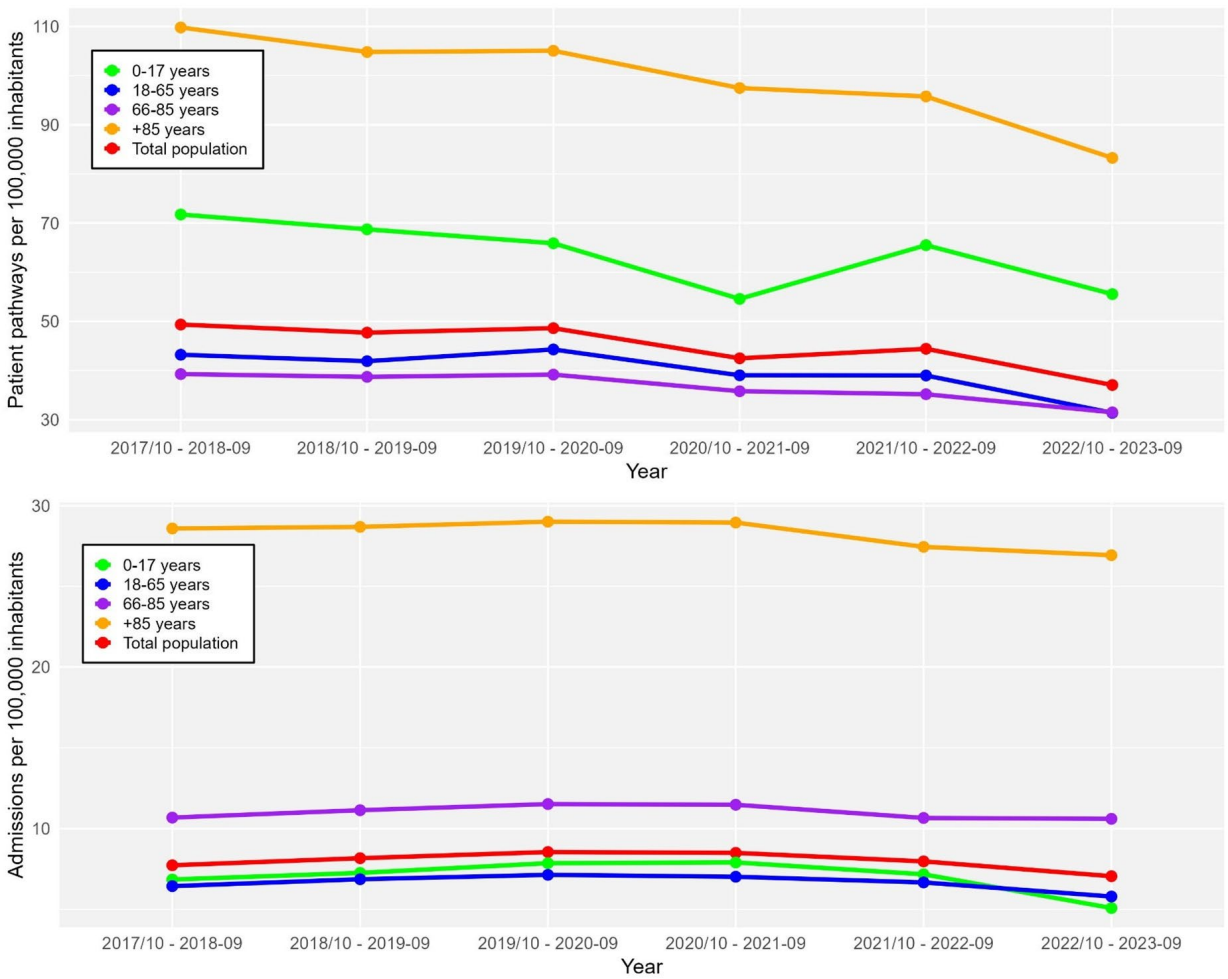
Incidence rates of patient pathways and admissions. This figure illustrates the temporal trends in incidence rates of patient pathways to the out-of-hours service (top) and admissions resulting from these patient pathways (bottom), measured per 100,000 inhabitants across age groups from 2017 to 2023. The age groups represented are ‘0–17 years,’ ‘18–65 years,’ ‘66–85 years,’ ‘+85 years,’ and ‘Total.’ The incidence rates are displayed for each year period. The upper plot shows the overall incidence of patient pathways, while the lower plot focuses on the subset that resulted in hospital admissions. Each colored line represents a specific age group, and the ‘Total’ is shown separately for comparison.

Approximately half of all patient pathways occurred on weekdays (GP-OOHS: 47.4%, 1818: 47.8%). Most patient pathways took place in the early evening (16:00–22:00), with 54.6% in GP-OOHS and 52.2% in 1818. Weekend daytime patient pathways (08:00–15:00) were largely equivalent between GP-OOHS and 1818 (31.0% vs. 30.3%), while night-time patient pathways (23:00–07:00) were marginally lower in GP-OOHS (15.2% vs. 16.8%). Throughout the study period, the total number of patient pathways decreased from 412,116 in 2017 to 314,879 in 2023. ED admissions following OOHS patient pathways also declined, from 64,555 admissions in 2017 to 59,967 in 2023 equivalent to 15.7% and 19.0% of pathways, respectively. In the interrupted time series analysis using 12-month periods (October–September), the number of pathways showed a continuous annual decline of approximately 3% prior to the organisational transition to the 1818, followed by an additional 12% level decrease after October 2022 (*p* < 0.001). Although the crude proportion of admitted patients increased over time, the binomial model indicated that the odds of admission decreased by about 6% relative to the pre-intervention trend (*p* < 0.001), suggesting that the previously increasing tendency in ED admissions flattened following the transition. (Table 3, Figure 3). Among children (0–17 years), the proportion of patient pathways resulting in admission rose steadily in the early years of the study: from 9.6% in 2017 to 10.6% in 2018, 11.9% in 2019, and 14.5% in 2020. After the organizational shift to 1818, admission rates declined to 11.0% in 2022 and reached 9.2% in 2023. This decline coincided with a marked reduction in the number of paediatric patient pathways over time.

**Table 3.** Annual trends in hospital admissions by age group.

	2017/10–2018–09	2018/10 − 2019-09	2019/10 − 2020-09	2020/10 − 2021-09	2021/10 − 2022-09	2022/10 − 2023-09
Patient pathways, all ages, (n)	412,116	399,212	407,052	356,419	374,686	314,879
Incidence per 100,000 inhabitants	49,354	47,711	48,611	42,490	44,420	37,051
Admitted within 24 h (n)	64,555	68,364	71,594	71,293	67,253	59,967
Proportion admitted (%)	15.7%	17.1%	17.6%	20.0%	17.9%	19.0%
Patient pathways, age 0–17, (n)	119,556	113,357	107,733	88,818	106,591	90,373
Incidence per 100,000 inhabitants	71,749	68,731	65,888	54,572	65,504	55,539
Admitted within 24 h (n)	11,418	11,974	12,853	12,869	11,674	8,277
Proportion admitted, (%)	9.6%	10.6%	11.9%	14.5%	11.0%	9.2%
Patient pathways, age 18–65, (n)	213,526	207,059	218,255	192,067	192,634	156,155
Incidence per 100,000 inhabitants	43,210	41,900	44,283	39,023	38,989	31,382
Admitted within 24 h (n)	31,829	33,938	35,19	34,563	32,964	28,854
Proportion admitted (%)	14.9%	16.4%	16.1%	18.0%	17.1%	18.5%
Patient pathways, age 66–85, (n)	62,512	62,881	64,836	60,17	59,999	54,421
Incidence per 100,000 inhabitants	39,269	38,708	39,167	35,786	35,175	31,490
Admitted within 24 h (n)	17,005	18,095	19,069	19,296	18,182	18,329
Proportion admitted (%)	27.2%	28.8%	29.4%	32.1%	30.3%	33.7%
Patient pathways, age +85, (n)	16,522	15,915	16,228	15,364	15,462	13,930
Incidence per 100,000 inhabitants	109,803	104,814	105,063	97,481	95,764	83,259
Admitted within 24 h (n)	4,303	4,357	4,482	4,565	4,433	4,507
Proportion admitted (%)	26.0%	27.4%	27.6%	29.7%	28.7%	32.4%

There is a consistent decline in the total number of patient pathways and those not admitted over the years across all age groups. Despite fewer overall patient pathways, the proportion of patients admitted within 24 h increased, particularly among older age groups. The decrease in the number of children admitted to emergency department corresponds with a higher admission proportion, reflecting a more selective approach to hospital referrals, focusing on cases with higher medical necessity. The overall reduction in admissions and the increased admission proportion suggest a shift towards managing higher acuity cases.

In adults aged 18–65 years, the total number of patient pathways decreased from 213,526 in 2017 to 192,634 in 2022 and further to 156,155 in 2023 equivalent to a 27% reduction over the period. Despite this decline in patient pathways, the proportion admitted increased throughout most of the period, reaching 18.5% in 2023. However, the absolute number of admissions in this group fell to 28,854 by 2023, the lowest level observed during the study period.

Among patients aged 66–85 years, a similar decline in patient pathways was observed, particularly after the transition to the 1818. Patient pathways fell from 62,512 in 2017 to 54,421 in 2023, while the proportion admitted increased to 33.7% (18, 329) in 2023 compared to 30.3% (18, 182) in 2022 and 27.2% (17,005) in 2017.

For patients aged 85 years and older, the total number of patient pathways decreased from 15,462 in 2017 to 13,390 in 2023. Simultaneously, the number of admissions increased slightly from 4,433 (28.7%) in 2017 to 4,507 (32.4%) in 2023.

Across all age groups, incidence rates of patient pathways to OOHS declined over the study period, while incidence rates of ED admissions showed a less pronounced decline (Figure 3). Seasonal variations and the impact of the COVID-19 pandemic were also observed. In particular, a reduction in face-to-face consultations occurred during the pandemic period, followed by a return to pre-pandemic levels by 2022 under 1818 (Supplementary Figure 1).

## Discussion

In this retrospective cohort study of patients contacting OOHS in Region Zealand from January 2017 to October 2023, we analysed a dataset of 2,572,951 patient pathways. A decline in patient pathways and subsequent ED-admissions was found, and the decline was stable including last year when the organisation transitioned from GP-OOHS to 1818.

Over the study period, the annual number of patient pathways declined from 412,116 to 314,879, and subsequent ED admissions decreased from 64,555 to 59,967. The reduction in patient pathways predominantly affected patient categories that did not result in ED admission. Age-specific patterns showed a decline among the youngest patients, a moderate decrease among middle-aged groups, and relatively stable admission rates among the oldest patients. In the last year of the study period where the 1818 was operated as an integrated service, a larger proportion of the patients were admitted to the ED, though this in absolute numbers was lower than in any of the previous years. For comparison, there were a total of 281,577 ED admissions in Region Zealand in 2023.

The transition from GP-OOHS to 1818 is part of a broader trend, where OOHS mainly operated by GPs is in decline internationally [11]. While several studies have described the influx of patients in OOHS [12] , little is known about the impacts of new systems in treating medical conditions outside office hours. In a European setting, Danish OOHS are known to be amongst the most visited OOHS [13]. In a previous study, Huibers et al. found that Danish OOHS had 481 crude contacts per 1,000 personyears, which was found to be higher than comparative system [14]. We investigated several consecutive years (2017–2023), allowing us to investigate the use of OOHS over time.

The Danish EDs have seen an overall increasing level of activity, and especially The Capital Region of Denmark have shown an increasing incidence rate of contacts, after the organisational change of the OOHS to an integrated organisation [15]. Such an increase could be expected in The Capital Region, where patients are seen at hospital-based facilities, and it has given rise to some debate regarding its potential impact on ED workload. While the concern is very real and was voiced prior to the transition from GP-OOHS to 1818 in October 2022, in this study the actual number of admitted patients decreased in absolute numbers, while the proportion of OOHS patient pathways with a subsequent admission increased. Further, in the interrupted time series analysis, we found that the transition towards an integrated organisation appeared to slow down the previous upward trend in the proportion of contacts resulting in admission, even though the overall proportion of admissions was higher. Preliminary observations from an ongoing investigation may offer some context for these developments. Early analyses of patients’ contact patterns prior to admission indicate a shift in help-seeking behaviour during the study period. Specifically, we observe indications of an increasing proportion of patients contacting the emergency medical number 1-1-2 rather than OOHS before admission. Moreover, we found a rise in patients being admitted without any preceding contact. These findings are unpublished and not yet peer-reviewed, and should therefore be interpreted with caution. Nevertheless, they may reflect broader changes in patient behaviour that could contribute to the observed decline in OOHS pathways and admissions.

## Limitations

This study compares patient pathways in OOHS during Cooperative GP and 1818 from January 2017 to October 2023. The dataset is asymmetrically composed, with approximately 87% of contacts originating from *GP-OOHS* and 13% from 1818. This imbalance reflects the relative duration of each organizational period but may nonetheless limit the precision of comparative analyses. While the system used to record patient contacts and services was unchanged, there might have been differences in how patient pathways and underlying services were recorded, as physicians in 1818 were not enumerated per service delivered and hence might not have been as rigorous in recording all delivered services. However, registration was generally thorough across both organizational models. In the GP-OOHS, financial incentives may have encouraged more detailed service-level entries, whereas in 1818, structured registration procedures ensure consistent documentation. All patient contacts were required to be linked to at least one service code. Although the same coding rules applied in both systems, minor deviations cannot be excluded. By analysing complete patient pathways rather than individual services, the risk of differential under- or overcounting is minimized, as each pathway necessarily includes an initial registered service. However, we cannot exclude that call-backs within the same pathway may have contributed additional clinical information that is not fully captured in our pragmatic linkage of admissions to the highest-ranking interaction. These differences could influence the number of individual services but are unlikely to affect the integrity of patient pathway tracking.

Secondly, we have enriched the OOHS dataset with information on subsequent hospitalization. This is taken from the national patient database, and while the ED contact is within 24 h from the last contact to OOHS, the two contacts might not be related, though this is most likely. There is no referral information in the national patient database, so in this study, we assume the two contacts are linked, if admission appear within 24 h.

Finally, we have not had access to waiting time on calls to OOHS during the study period. It is known, that extended waiting times for OOHS can result in patients seeking out alternatives such as self-referral to ED or using emergency medical services [16]. This might result in a bias, which has not been investigated in this study.

## Conclusion

In this retrospective cohort study of OOHS patient pathways in Region Zealand, Denmark, we investigated the effect of the new organization on admission to EDs. We found an overall decline in patient pathways in OOHS during the entire period and continuing after the organizational change. We found the decline in both the number of patient pathways in OOHS and the absolute number of patients admitted to an ED. However, the relative proportion of pathways resulting in ED admission increased slightly following the organizational change. These findings indicate a continued decline in OOHS activity and a slight shift toward more acute cases after the reorganisation.

## Supplementary Material

Supplemental Material

## Data Availability

The data that support the findings of this study are not openly available due to reasons of sensitivity and are available from the corresponding author upon reasonable request.
