# Supplementary material for: Out-of-hours services in Zealand, Denmark. Consequences of changeover from GP-cooperative to integrated deputized services. A retrospective cohort study
Source: Scand J Prim Health Care. 2026 Feb 7;44(1):2616519. doi: 10.1080/02813432.2026.2616519 (PMC12885031; doi:10.1080/02813432.2026.2616519)
Supplement: Supplemental Material [file IPRI_A_2616519_SM7243.docx]

**Supplementary:**


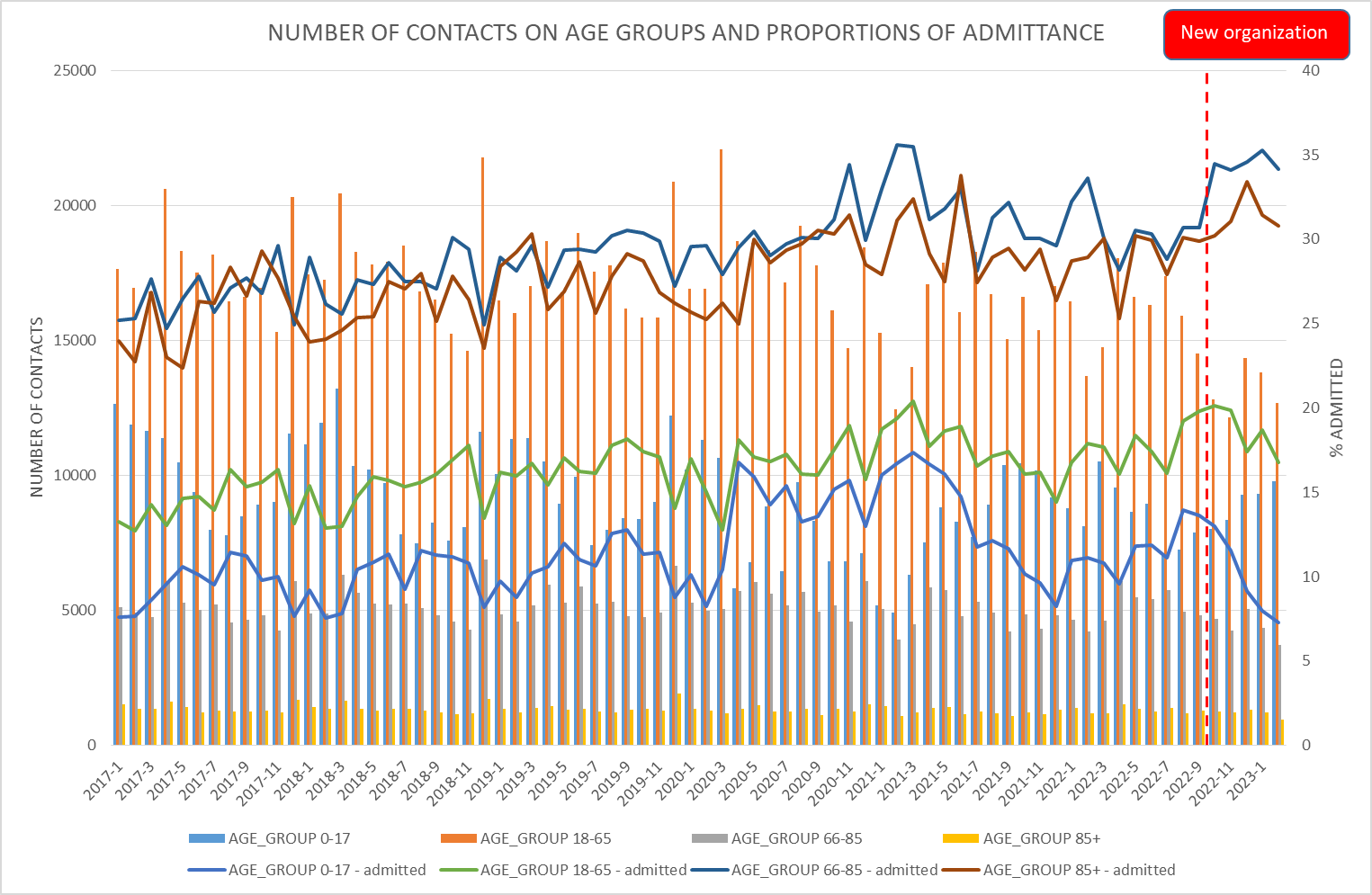
Figure 1: Number of contacts on age groups and proportions of admittance in Region Zealand during the period from 2017-2023.

**0-17 Years Age Group**

- - The chart shows fluctuating contact volumes with periodic peaks, possibly indicating seasonal health issues affecting this age group.
  - A rising trend in admission rates after the introduction of the new organization is observed, with variations in rates across the timeline.

**18-65 Years Age Group (Second Chart):**

- - A higher volume of contacts is noticeable compared to the 0-17 age group, with the admission rate line indicating a less pronounced upward trend post-transition.
  - This demographic represents the working-age population, and the admission rates may correlate with occupational health trends or broader health policy impacts.

**66-85 Years Age Group (Third Chart):**

- - The contact volume is less than the 18-65 group but shows greater stability over time.
  - There is a significant increase in the admission rate line post-transition, potentially due to this age group's more complex health needs and the new organization's operational policies.

**85+ Years Age Group (Fourth Chart):**

- - The contact volumes are the lowest among all groups, reflecting the smaller population size of this demographic.
  - The admission rates for this group are the highest, which is consistent with the age-related increase in healthcare needs. Post-transition, the chart suggests a slight trend towards increased admissions, possibly due to enhanced focus on acute care needs for the oldest patients.

Figure 2: Monthly Admission Rates by Consultation Type for PLO and RSJ in Region Zealand OOHS

**Home Visits**: For PLO, the admission rates for home visits (blue solid line) show substantial variability, peaking around December. In contrast, RSJ (blue dashed line) maintains a higher admission rate for home visits, which remains relatively constant over the months, indicating a consistent policy or patient acuity that leads to admissions after such visits.

**Face-to-Face Consultations**: The admission rates following face-to-face consultations (orange lines) are similar for both models but generally higher in RSJ. This might suggest that face-to-face assessments in RSJ tend to lead to hospital admissions more often than in PLO.

**Telephone Consultations**: Admission rates following telephone consultations (grey lines) are consistently lower for PLO compared to RSJ. This trend could reflect a more conservative approach in the RSJ model or differences in patient conditions that require follow-up care.

**Video Consultations**: Admission rates after video consultations (yellow lines) are the lowest among all consultation types for PLO and exhibit slight fluctuations for RSJ. This suggests that conditions managed through video consultations in both models are less likely to require immediate admission.

Figure 3: Comparison of Monthly Hospital Admission Rates After Home Visits by PLO and RSJ

**Trends Over Time:** The graph shows monthly percentages, with each provider represented by a distinct color line—PLO in orange and RSJ in green.

**Seasonal Variations:** There appears to be a seasonal variation in admission rates for both providers, with fluctuations across months. However, the patterns of these fluctuations differ between the two providers.

**Comparative Admission Rates:** The RSJ (green line) generally exhibits a lower percentage of admissions after home visits across most of the year compared to PLO (orange line), except for a notable peak around March where RSJ surpasses PLO.

**Interpretation:** The higher overall admission rates for PLO could indicate a more direct approach to patient management, where home visits are more likely to result in hospital admissions. This could reflect a model of care that is more inclined to escalate care when deemed necessary. Conversely, RSJ's generally lower admission rates might suggest more conservative criteria for hospital admission from home visits or possibly more effective in-home care interventions that prevent the need for hospitalization.

Figure 4: Monthly Admission Rates After Face-to-Face Consultations for PLO vs. RSJ

**Orange Line (PLO):** This line shows the proportion of admissions for the PLO over the year. It starts just above 20% in October, dips slightly in November, then peaks above 20% in December before gradually declining and leveling off. There is another peak around July before it stabilizes for the rest of the period.

**Green Line (RSJ):** The RSJ line begins below 15% in October, showing a lower admission rate than PLO. It sees a significant rise in November, surpasses the PLO in December, and generally follows an upward trajectory until March. After a slight dip, it rises again and remains relatively steady, ending the period slightly below the PLO admission rates.

**Seasonal Trends:** The graph indicates possible seasonal trends, with admission rates peaking in the winter months for both organizations, which could correspond to increased prevalence of seasonal illnesses.

**Comparative Analysis:** The PLO generally maintains higher admission rates after face-to-face consultations compared to RSJ, except for a notable period between November and March when RSJ admissions surpass those of PLO. This could suggest differences in the thresholds for hospital admissions between the two models or variability in the acuity of cases presented to each service.

Figure 5: Face-to-Face Consultation Admission Rates: PLO vs. RSJ Over a Year. The proportion of admissions for PLO is depicted by an orange line, while admissions for RSJ are represented by a green line.

- **Admission Trends:** The admission rates for PLO show a peak around December, dipping in the following months and then gradually rising towards September. For RSJ, the rates start lower in October, see a rise in December, dip in the ensuing months, and climb back up, finishing the period slightly below the PLO rates.
- **Seasonal Variability:** Both lines suggest potential seasonal influences, with higher admission rates during the winter months which could correlate with seasonal health conditions that require face-to-face consultations.
- **Comparative Analysis:** The PLO tends to have higher admission rates than RSJ, except for a few months where RSJ's rates approach or equal those of PLO. This could reflect differences in operational practices, patient case severity, or triage processes between the two healthcare providers.

Figure 6: Yearly Trends in Hospital Admissions After Video Consultations for PLO vs. RSJ

**Orange Line - PLO (Cooperative GP):** The PLO’s admission rates after video consultations start moderately in October and exhibit a significant peak in December, reaching just over 10%. This peak suggests a higher likelihood of admissions during this period, possibly due to winter-related illnesses that are not sufficiently addressed through remote consultations. Following this peak, there is a noticeable decrease in January, and the rates stabilize somewhat from February to May, oscillating between approximately 8-9%. However, there is another rise observed in June, which remains fairly consistent through August before experiencing a slight decline in September.

**Green Line - RSJ (Integrated organization):** The RSJ starts off the period with a lower admission rate in October compared to PLO. It sees an increase in November, surpassing the PLO, and reaches its peak in December, similar to the PLO. This could indicate a common seasonal effect on health conditions requiring hospital admission post-consultation for both service models. RSJ’s rates then descend and remain below PLO’s until a rise in June that continues to ascend modestly, ending the period at around 9%, slightly lower than PLO’s rate.

**Seasonal Variations and Trends:** The graph indicates potential seasonal variations affecting both service models, with the winter months showing the highest admission rates. This could be due to the prevalence of seasonal illnesses that necessitate in-person care despite initial video consultations.

**Comparison Between Models:** Throughout most of the year, PLO’s admission rates are higher than RSJ's, which might suggest PLO's video consultations lead to hospital admissions more frequently. This could reflect differences in the criteria used to determine the need for hospitalization, the effectiveness of video consultations, or potentially the complexity of cases managed by each service model.
